# Supplementary material for: Depolymerization mechanisms and closed-loop assessment in polyester waste recycling
Source: Nat Commun. 2024 Jul 25;15:6266. doi: 10.1038/s41467-024-50702-5 (PMC11269573; doi:10.1038/s41467-024-50702-5)
Supplement: Supplementary file 1 — SI [file 41467_2024_50702_MOESM1_ESM.pdf]

Supplementary Information

Depolymerization mechanisms and closed-loop assessment in polyester waste recycling

Jingjing Cao[#,1], Huaxing Liang[#,1], Jie Yang[2], Zhiyang Zhu[1], Jin Deng[*,2], Xiaodong Li[*,3], Menachem Elimelech[*,4], and Xinglin Lu[*,1]

[1] CAS Key Laboratory of Urban Pollutant Conversion, Department of Environmental Science and Engineering, National Synchrotron Radiation Laboratory, University of Science and Technology of China, Hefei 230026, China.

[2] CAS Key Laboratory of Urban Pollutant Conversion, Anhui Province Key Laboratory of Biomass Clean Energy, Department of Applied Chemistry, University of Science and Technology of China, Hefei 230026, China.

[3] Max Planck Institute of Microstructure Physics, Weinberg 2, Halle 06120, Germany.

[4] Department of Chemical and Environmental Engineering, Yale University, New Haven, Connecticut 06520-8286, USA.

[#] These authors contributed equally.

* Corresponding authors
Email: xinglinlu@ustc.edu.cn (X. Lu); menachem.elimelech@yale.edu (M. Elimelech); xiaodong.li@tu-dresden.de (X. Li); dengjin@ustc.edu.cn (J. Deng)

Table of contents

Supplementary methods

Supplementary figures

Supplementary tables

Supplementary methods

Materials and chemical reagents. In all experiments, PET fragments were obtained from various PET products, including transparent and colored post-consumer PET lunch boxes and trays, PET water bottles, as well as PET textiles. Key chemicals including zinc chloride anhydrous, L-alanine, ferric chloride, ethanolamine, and ethylene glycol antimony were procured from Sigma-Aldrich. Sodium hydroxide, methanol, and ethanol were received from Sinopharm Chemical Reagent. All chemical reagents were used as received, without any further purification.

Evaluation of polymerization performance. A standard polymerization process comprises the following steps. First, DMT (50 g), EG (19.2 g, 1.2 eq), and ethylene glycol antimony (25 mg, 0.05 *wt*%) were mixed in a 250 mL custom-made titanium polyester reactor. The reactor was purged with N_2 five times to create an inert atmosphere. The transesterification reaction occurred at temperatures ranging from 180 to 190 °C. The progress of the reaction was tracked by measuring the amount of methanol released as a by-product. Following a duration exceeding 4 hours, the transesterification reaction was deemed complete. Subsequently, the polycondensation reaction was initiated at 270 °C under a stringent high vacuum of less than 100 Pa. Comprehensive characterization of the polymer's structure and properties was achieved through techniques including proton nuclear magnetic resonance (^1H NMR), differential scanning calorimetry (DSC), and gel permeation chromatography (GPC).

Catalyst regeneration conditions. The reacted catalyst was stirred in hot ethanol (70 °C) for 20 minutes at 300 rpm, then filtered to collect the filter cake. The cake was dried at 60 °C for 8 hours and subsequently transferred to a tube furnace for calcination at 350 °C for 1 hour in an atmosphere of 1 *vol* H_2 /99 *vol* N_2, with a heating rate 2 °C /min.

High-performance liquid chromatography (HPLC) analysis: The glycolysis

procucts were analyzed using an Agilent 8860 HPLC system equipped with a C18 column and an ultraviolet (UV) detector set at 254 nm. A 50:50 (v/v) methanol/H_2O mixed solution was used as the mobile phase at a flow rate of 1.0 mL/min.

Gas chromatography (GC) analysis: The methanolysis products were analyzed using an Agilent 8860 GC system equipment with an HP-5 column (30 m × 0.25 mm). The column temperature was initially set at 50 °C (held for 5 minutes) and then increases to 250 °C (held for 5 minutes) at 10 °C·min^{-1}. An internal standard, *n*-heptane, was added to the solution and homogeneously mixed after the reaction.

Detection of the isotope tracing liquid products in PET conversion under various conditions. Investigation of isotope tracing in liquid products during PET conversion was conducted using synchrotron radiation single-photon vacuum ultraviolet photoionization mass spectrometry (SVUV-PIMS). These measurements were carried out on the Beamline BL04B at the National Synchrotron Radiation Laboratory (Hefei, China). Gas samples, extracted immediately following one-sun irradiation, were analyzed at a photon energy of 14.5 eV. The experimental conditions were delineated as follows: (1) A mixture of 5 mg Fe/ZnO and 50 mg **Mode 1** were dispersed in a solvent composed of 29 mL methanol (CH_3OH) and 1 mL deuterated methanol (CD_3OD). This mixture was subjected to a reaction at 160 °C for 1 hour. (2) A combination of 5 mg Fe/ZnO and 100 mg **Mode 1** catalyst were dispersed in 30 mL CH_3OH, undergoing the same reaction conditions as outlined in Condition (1). (3) A blend of 5 mg Fe/ZnO and 100 mg pure PET were dispersed in 29 mL CH_3OH and 1 mL CD_3OD, reacting at 160 °C for 1 hour. (4) A mixture of 5 mg Fe/ZnO and 100 mg pure PET were dispersed in 30 mL CH_3OH, under the identical conditions as specified in Condition (1).

***In situ* attenuated total reflectance (ATR) infrared spectrometric experiments.** The structural evolution of the **Fe/Zn precursor** was investigated using *in situ* FTIR measurements. The experiments were carried out on a Bruker Vextex 70 spectroscope, which is equipped with a mercury-cadmium-telluride (MCT) detector. The

experimental setup allowed for precise control over the reactor temperature, which was increased from ambient conditions to 350 °C at a steady rate of 5 °C min^{-1}. Once the target temperature was reached, it was consistently maintained for 2 hours. During this thermal process, infrared spectra were captured at regular intervals of 10 minutes. The scans were acquired in the wavenumber range of 4000–600 cm^{-1} and all results were reported as the average of 64 individual scans.

***In situ* high-temperature and high-pressure infrared spectrometric experiments.** To elucidate the depolymerization processes of **Modes 1** and **2**, *in situ* FTIR measurements were carried out using a Bruker Vextex 70 spectroscope equipped with a mercury-cadmium-telluride (MCT) detector. First, the sample was pretreated in air flowing (20 mL min^{-1}) at 30 °C for 10 min. Once the spectra stabilized, a background measurement was recorded to establish a baseline for subsequent measurements. Following this, either CH_3OH or CD_3OD, each in a volume of 50 mL, was introduced into the reactor. the temperature was then incrementally increased from room temperature (~25 °C) to 160 °C at a controlled ramp rate of 10 °C min^{-1}. Upon reaching 160 °C, this temperature was sustained for 60 minutes. Typical signals indicative of intermediates formed during the depolymerization processes were diligently captured and analyzed using FTIR spectroscopy.

Details of DFT calculation. The first-principles calculations were conducted using the Vienna ab initio simulation package (VASP)[1, 2]. The interaction between ions and valence electrons is described using projector augmented wave (PAW) potentials, and the exchange-correlation between electrons is treated by using the generalized gradient approximation (GGA) in the Perdew-Burke-Ernzerhof (PBE) form[3]. The DFT-D3 method was employed to determine the van der Waals (vdW) interaction[4]. For rigorous simulations, the plane wave cutoff energy was set to 450 eV. G-center k-point meshes of 5 × 5 × 5 were used for bulk optimization. The ionic relaxations were carried out under the conventional energy (10^{-6} eV) and force (0.01 eV/Å) convergence criteria. This process determined the optimized lattice parameters of ZnO bulk as a = b = 3.21

Å, and c = 5.17 Å. Subsequently, a 3 × 3 × 1 supercell with an exposed [100] facet was constructed to simulate the pristine ZnO slab. The Fe-doping ZnO slab model is identified by replacing Zn atoms with Fe atoms at different positions. The G-center k-point meshes of 3 × 2 × 1 were used for the slab. Ionic relaxations were carried out under the conventional energy (10^{-5} eV) and force (0.01 eV/Å) convergence criteria. The theoretical approach is based on the GGA supplemented with an on-site Coulomb interaction parameter (GGA + U method), in which effective U-J parameters of 6.0 eV and 2.5 eV were applied to enhance the description of Zn 3d states and Fe 3d[5,6]. To avoid interactions between periodic images, a vacuum space of 15 Å was inserted in the z-direction. All calculations were performed under spin polarization conditions.

The formation energy of oxygen vacancy in the nanosheet slab was calculated by:

$$E_f = E(\text{vacancy}) + 1/2E(O_2) - E(\text{slab}) \quad (5)$$

where E_f denotes the formation energy of oxygen vacancy, and E(vacancy) is the calculated energy of the nanosheet slab with a vacancy. $E(O_2)$ and E(slab) represent the calculated energy of O_2 gas and pure nanosheet slab, respectively. For O_2 gas, $E(O_2)$ of -9.87 eV is obtained through a single point energy calculation[7]. Additionally, the climbing image nudged elastic band (CI-NEB) method was applied to evaluate the energy barriers of transition states (TS)[8, 9].

Comprehensive Life-Cycle Assessment (LCA) of closed-loop PET recycling. The depolymerization process was simulated using Aspen Plus V11 with the POLYSL model. We evaluated 18 indicators of the entire process (classified into three major categories including human health, ecosystems, and resources) using OpenLCA (Version, 1.10.3). Our system boundary encompasses a closed-loop recycling framework of PET, which includes the entire lifecycle from collection, and transportation to production, encompassing depolymerization and repolymerization, and the final extrusion into PET slices. We assessed the environmental impacts, specifically focusing on Non-renewable Energy Use (NREU) and Global Warming Potential (GWP) , across two distinct geographical regions: China and Europe. This comparative analysis provides a broader understanding of the environmental

implications in different global contexts. The chemical recycling processes were simulated on an industrial scale with an annual treatment of 200,000 tons of waste PET, using Aspen Plus V11 to obtain the mass balance and energy consumption. Further details on the data and assumptions applied in our LCA analysis are documented in the Supplementary Note 2 section.

Details of techno-economic analysis (TEA). The Aspen Process Economic Analyzer V11 was used to determine the capital and operating costs for conventional chemical plants. Discounted cash flow analysis was conducted, and the minimum selling price (MSP), defined as the selling price of the product when the net present value is zero, was calculated. Heat integration was implemented for the DMT monomers production using the Aspen Energy Analyzer (Aspen Technology 2019). Scenario analysis was performed on critical process parameters to account for uncertainties. Additional TEA assumptions are provided in the Supplementary Note 3 section.

The synthesis of *r*-PET *via* a transesterification-polymerization reaction. The polymerization was carried out after mixing DMT (1 equiv., 0.150 mol, 29.100 g) with ethylene glycol (2.4 equiv., 0.360 mol, 22.344 g) in a four-necked flask in the presence of 0.05 *wt.*% ethylene glycol antimony (Supplementary Fig. 1)[10]. The polymerization reaction underwent two steps: 1) transesterification and 2) solid-state polymerization (details provided in the Method section). The resulting product was analyzed using ^1H NMR spectroscopy (Supplementary Fig. 2). In particular, the disappearance of DMT–CH_3 protons at 3.8 ppm and the concomitant appearance of protons assigned to PET at δ = 4.8 ppm unambiguously verified the formation of *r*-PET[11]. Differential scanning calorimetry (DSC) analysis of the *r*-PET reveals a glass transition, a cold crystallization, and a melting transition temperature at 78, 190, and 245 °C, respectively[12], which are close to that of the pristine PET (75, 202, and 247 °C, respectively), see Supplementary Fig. 3. Moreover, the *r*-PET showed a molecular weight (M_n=58 *kDa*), which was only smaller than that of the pristine PET slice (M_n=64 *kDa*) (Supplementary Fig. 4). Taken together, these results demonstrate the effectiveness of using the obtained DMT monomers for achieving closed-loop recycling of PET wastes.

Supplementary figures

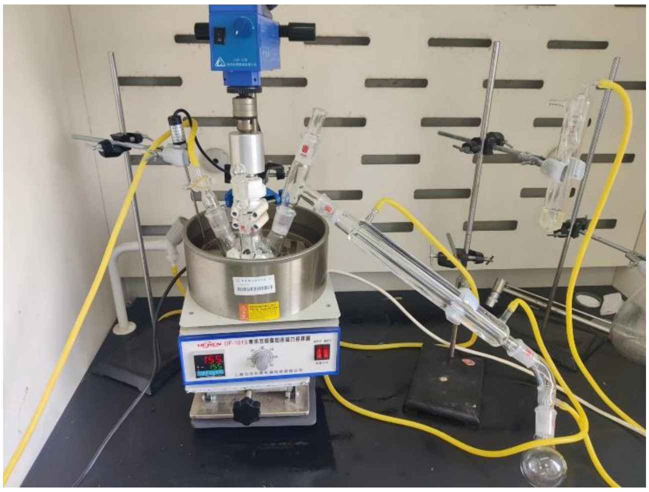

Supplementary Fig. 1. Setup used for polymerization of *r*-DMT to *r*-PET.

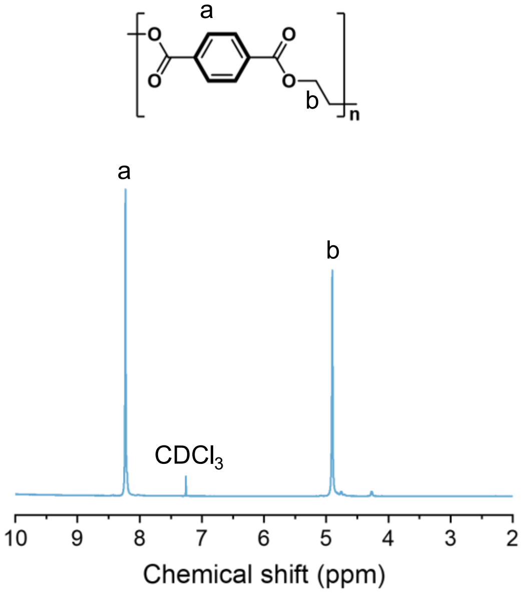

Supplementary Fig. 2. ^{1}H NMR spectrum of a *r*-PET sample recorded in $CDCl_3$/TFA (8/1).

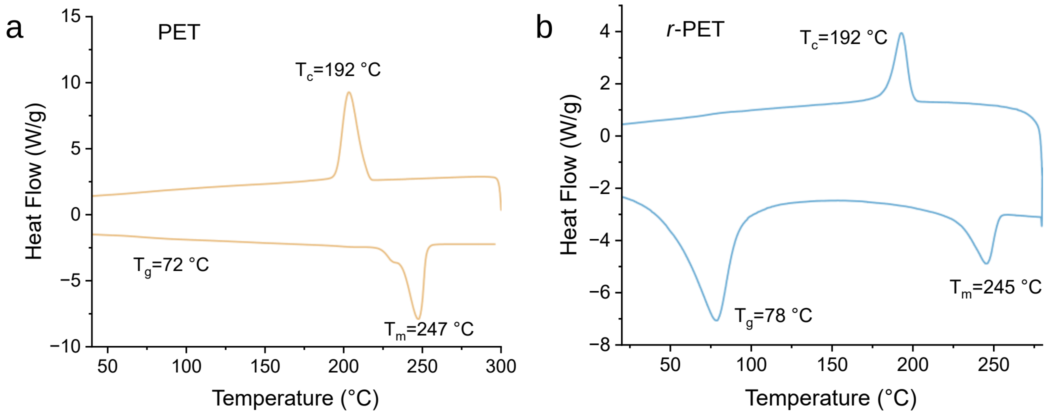

Supplementary Fig. 3. DSC profiles of PET and *r*-PET samples.

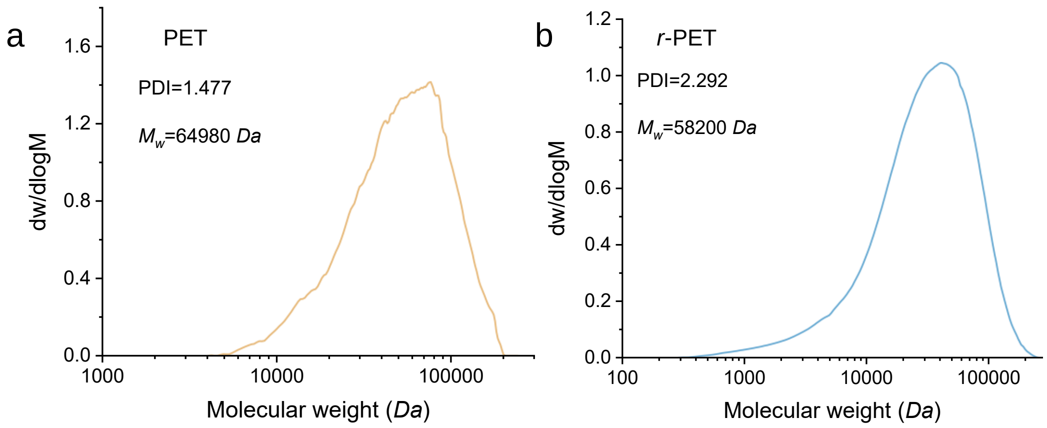

Supplementary Fig. 4. GPC profiles of PET and *r*-PET samples dissolved in hexafluoroisopropanol.

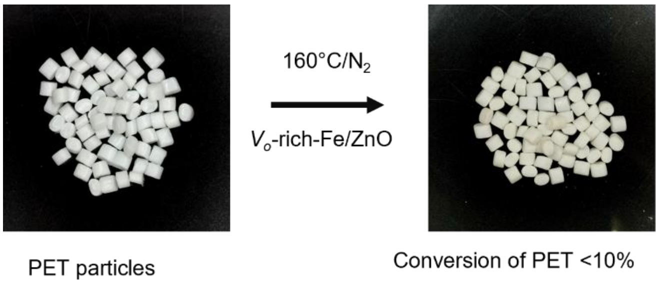

Supplementary Fig. 5. Photos of PET particles after the depolymerization reaction under the N_2 atmosphere.

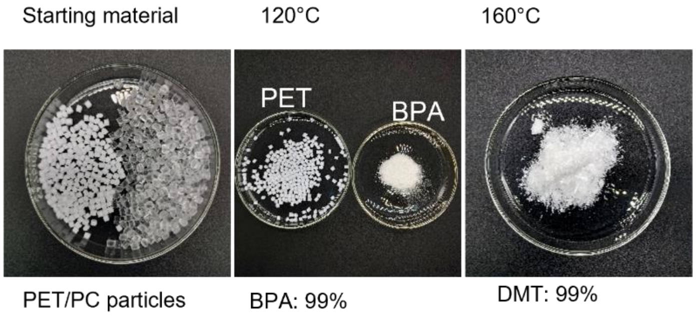

Supplementary Fig. 6. Photos of PET/PC mixed plastics through selective chemical depolymerization.

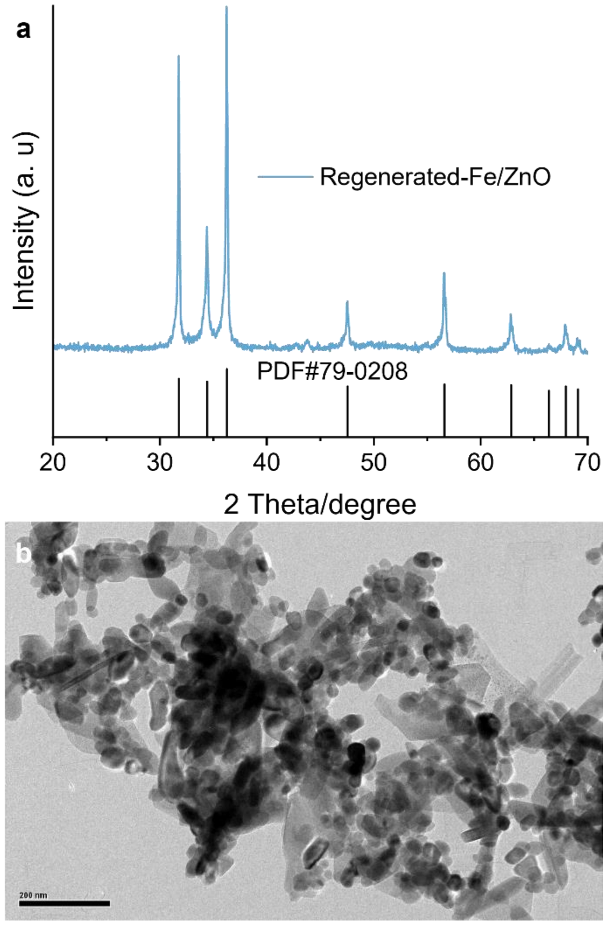

Supplementary Fig. 7. Phase and structure of the regenerated V_o-rich Fe/ZnO NSs after five catalytic cycles. **a** XRD pattern, and **b** TEM image.

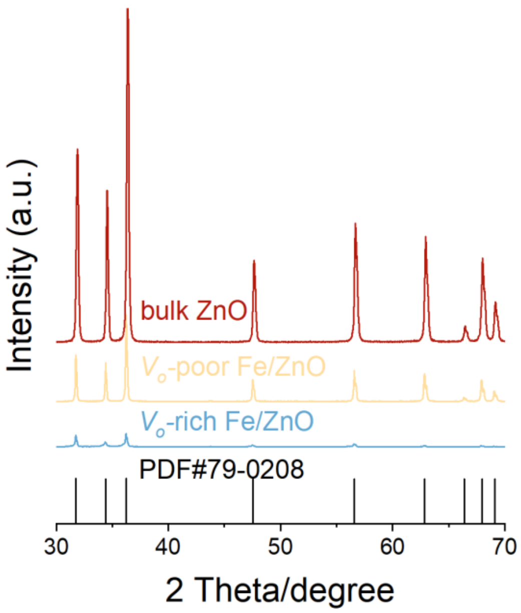

Supplementary Fig. 8. XRD profiles of bulk ZnO, V_o-poor Fe/ZnO NSs, and V_o-rich Fe/ZnO NSs.

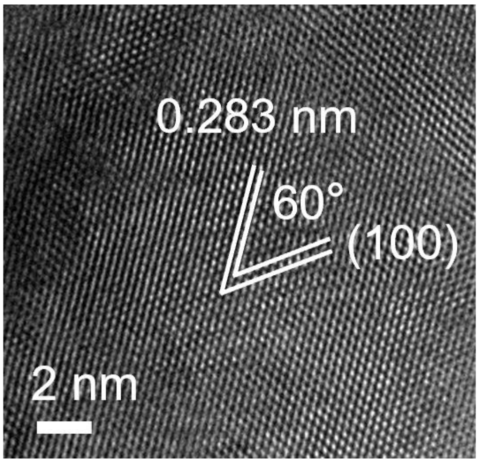

Supplementary Fig. 9. HRTEM image of V_o-rich Fe/ZnO NSs.

a

EDS

Element	Atomic Fraction (%)
O	52.6
Zn	46.7
Fe	0.7

ICP

Element	Atomic Fraction (%)
O	52.5
Zn	46.7
Fe	0.8

b

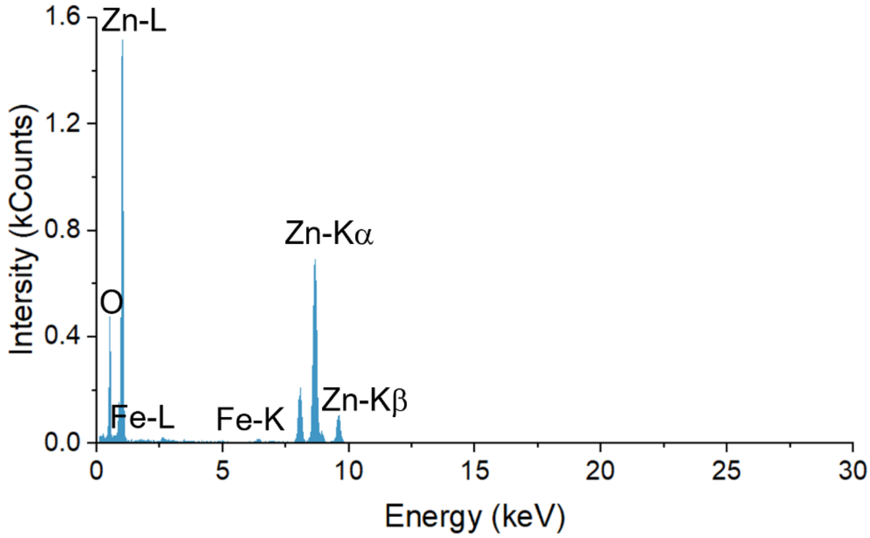

Supplementary Fig. 10. **a** EDS and ICP tables of V_o-rich Fe/ZnO NSs. **b** EDS spectrum of V_o-rich Fe/ZnO NSs.

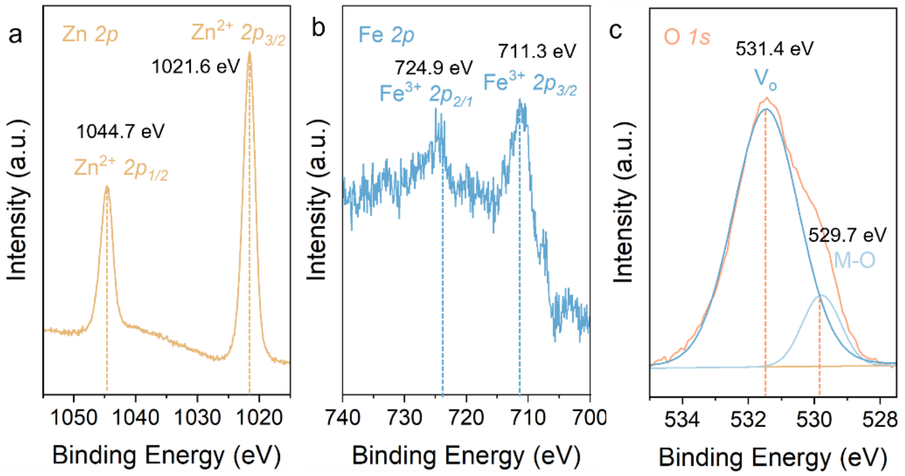

Supplementary Fig. 11. High-resolution XPS spectra of the as-prepared V_o-rich Fe/ZnO NSs catalysts. XPS spectra of **a** Zn *2p*, **b** Fe *2p*, and **c** O *1s*.

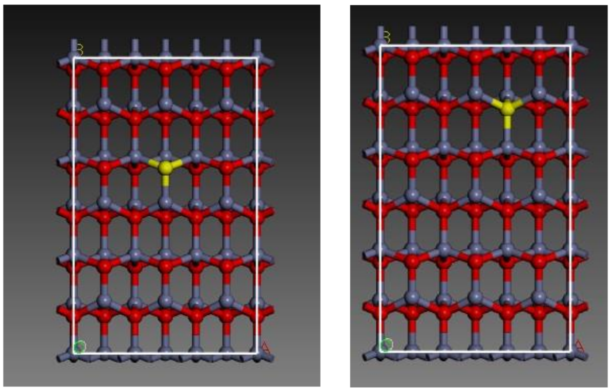

D1 D2

Supplementary Fig. 12. Optimized structures of D1 and D2 oxygen defects on ZnO (100) surface.

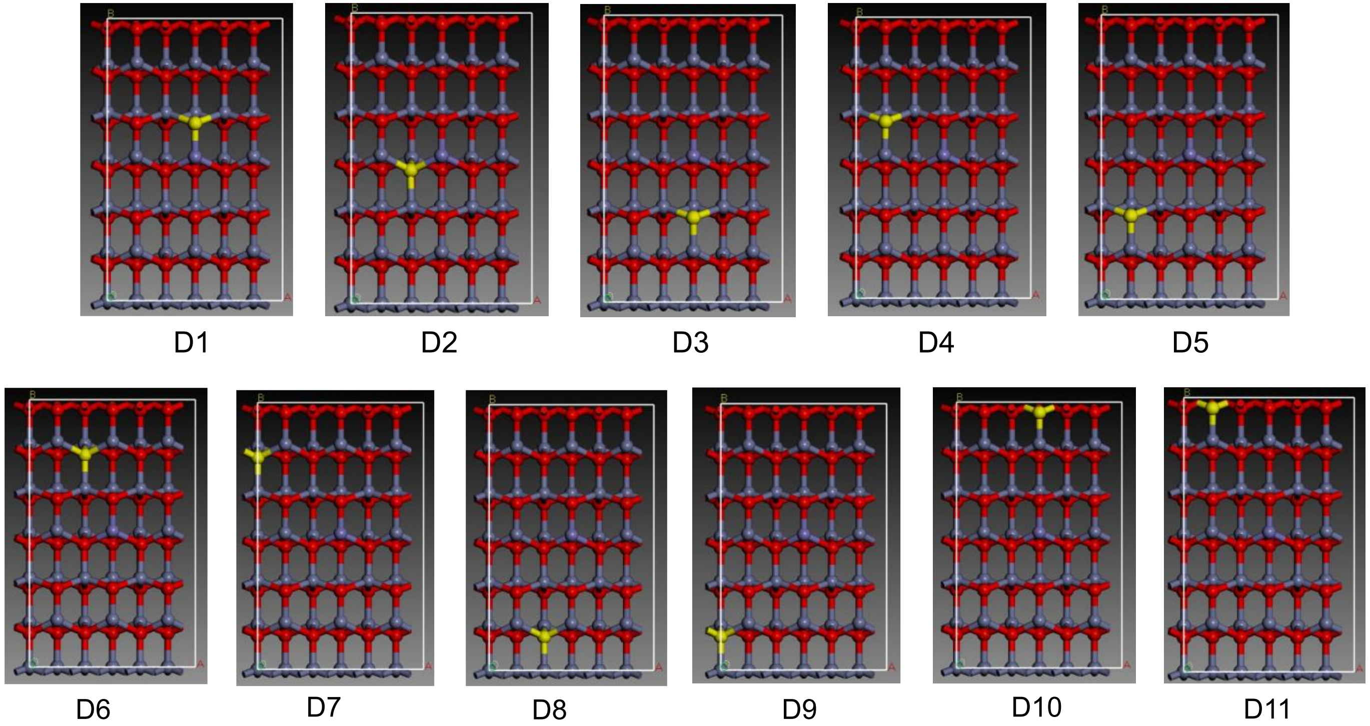

Supplementary Fig. 13. Optimized structures of D1, D2, D3, D4, D5, D6, D7, D8, D9, D10, and D11 oxygen defects on Fe/ZnO (100) surface.

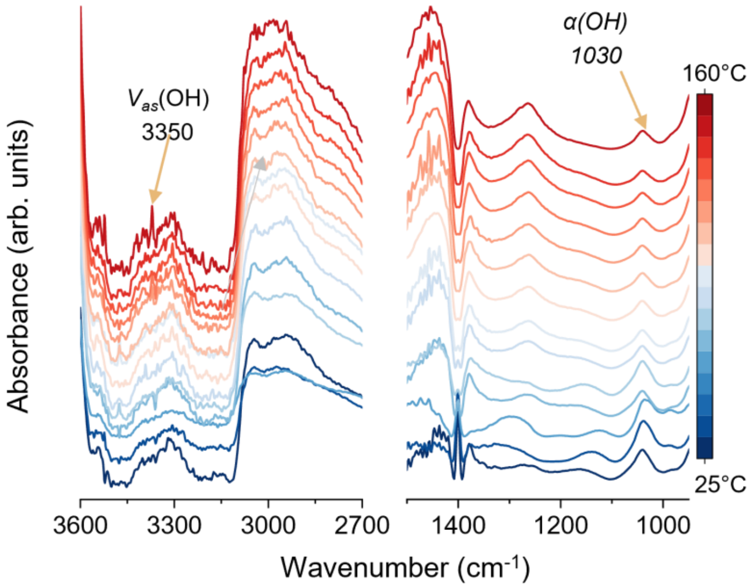

Supplementary Fig. 14. *In situ* attenuated total reflectance (ATR) infrared spectra of the change of methanol functional groups on V_o-rich Fe/ZnO NSs surface under the N_2 atmosphere.

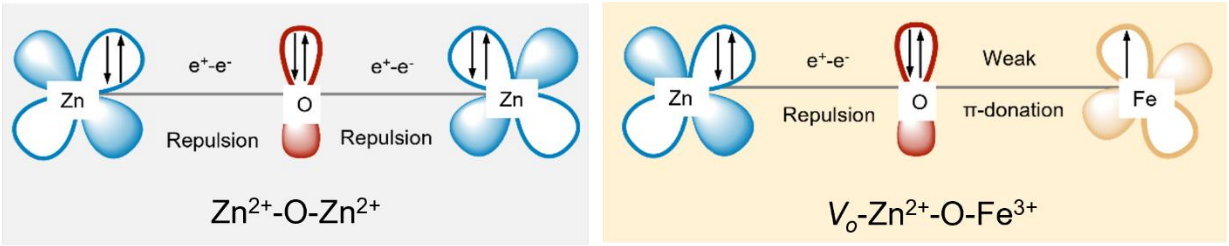

Supplementary Fig. 15. Schematic representations of the electronic coupling among bulk ZnO, and V_o-rich Fe/ZnO NSs.

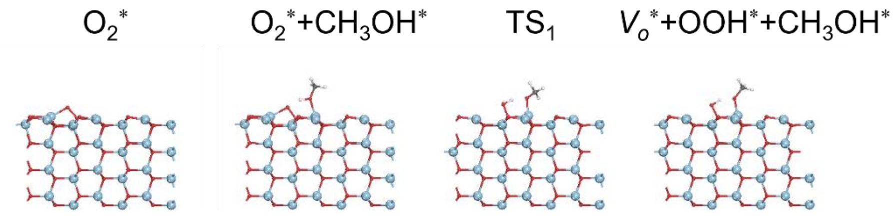

Supplementary Fig. 16. Changes of bulk ZnO slab in activating O_2 and methanol.

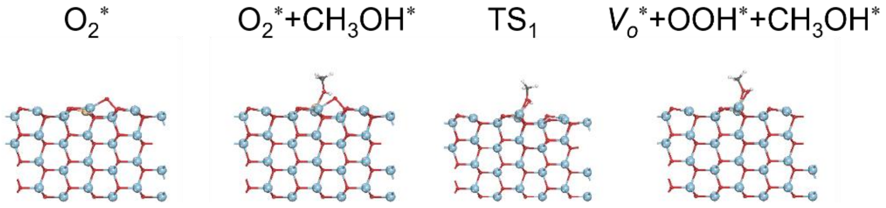

Supplementary Fig. 17. Changes of V_o-Fe/ZnO slab in activating O_2 and methanol.

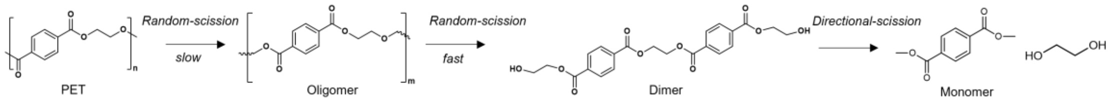

Supplementary Fig. 18. Different types of bond scission during PET depolymerization.

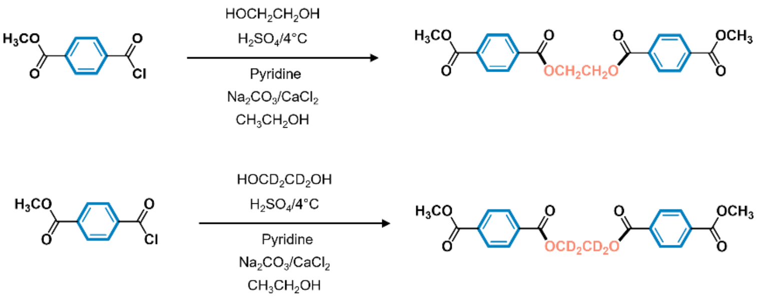

Supplementary Fig. 19. Synthetic routes of **Modes 1** and **2**.

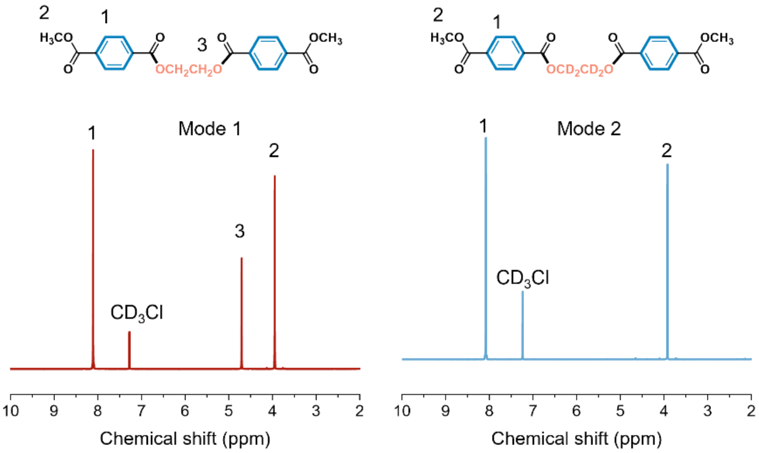

Supplementary Fig. 20. ^{1}H NMR spectra of **Modes 1** and **2** recorded in CD_3Cl.

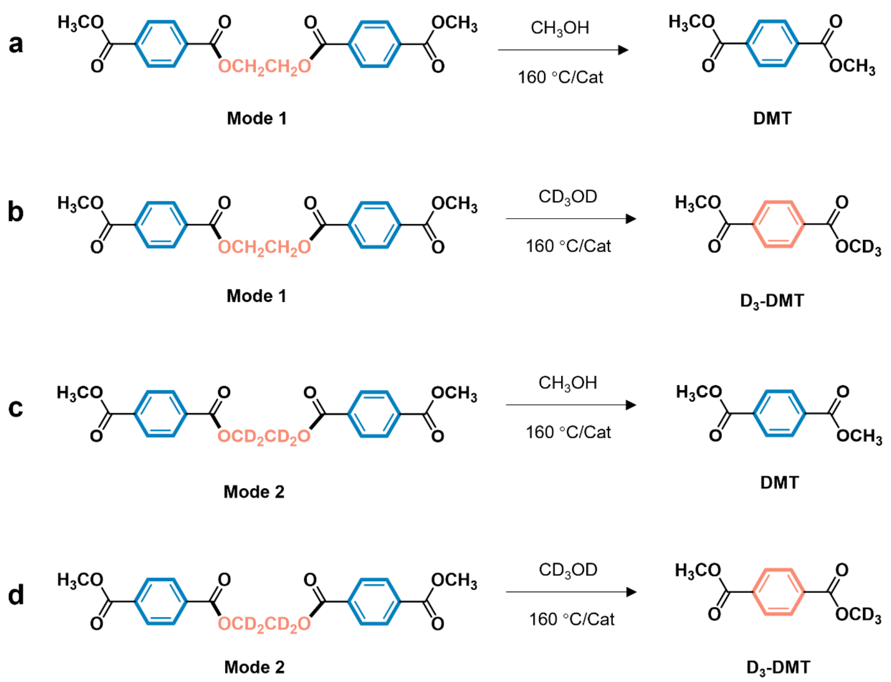

Supplementary Fig. S21. Methanolysis of **Modes 1** and **2** under methanol and methanol-*d*4.

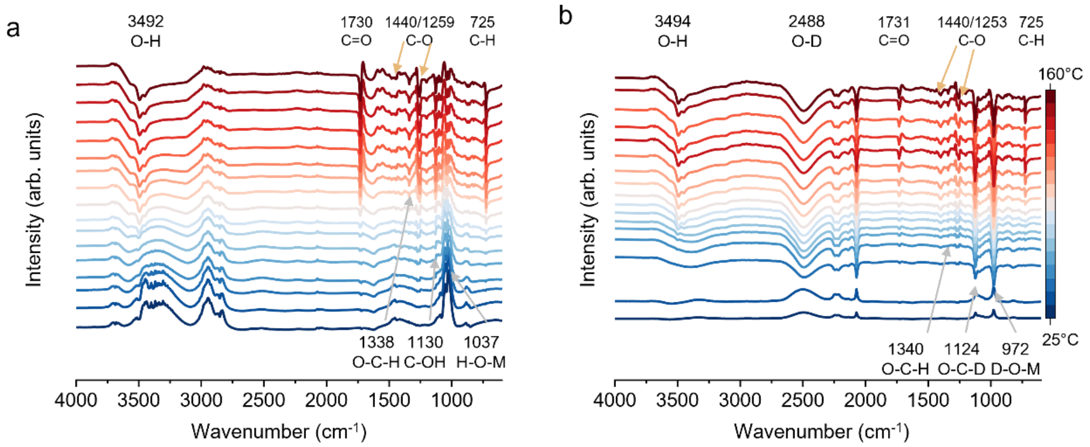

Supplementary Fig. S22. *In situ* high-temperature and high-pressure infrared spectrometry investigated by **Mode 1** depolymerization in (**a**) CH_3OH and (**b**) CD_3OD under air atmosphere.

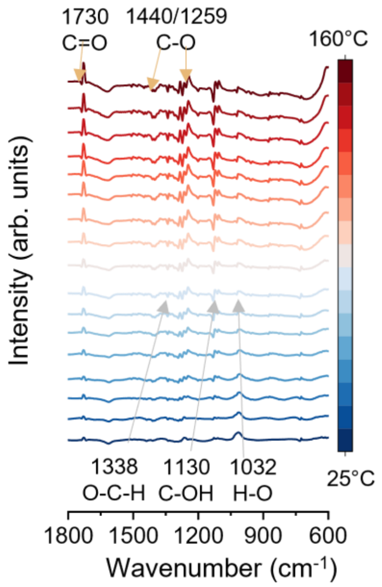

Supplementary Fig. S23. *In situ* high-temperature-pressure infrared spectrometry was investigated by **Mode 1** depolymerization under nitrogen atmosphere.

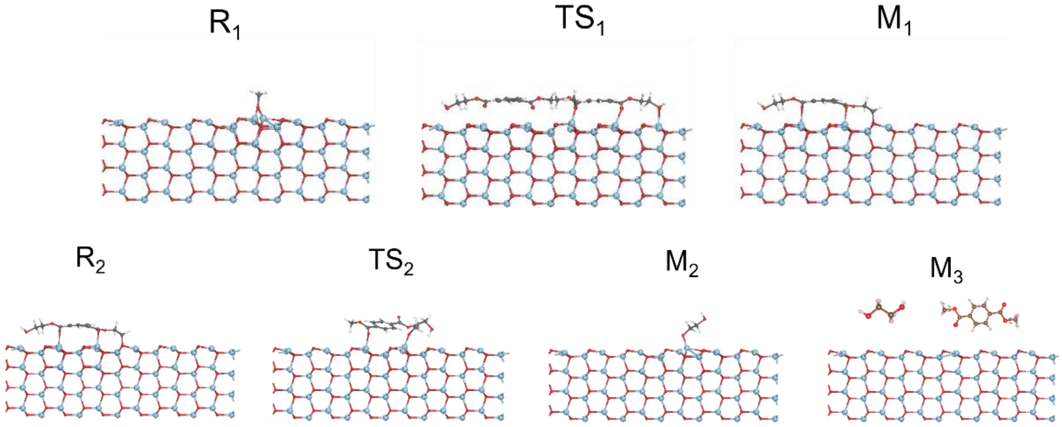

Supplementary Fig. 24. Change of bulk ZnO slabs in catalytic **Mode 1** depolymerization.

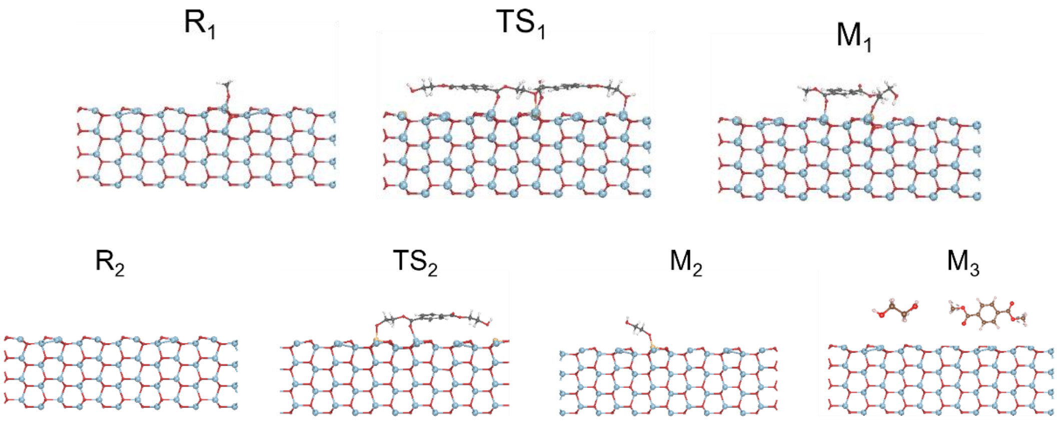

Supplementary Fig. 25. Changes of V_o-Fe/ZnO slabs in catalytic **Mode 1** depolymerization.

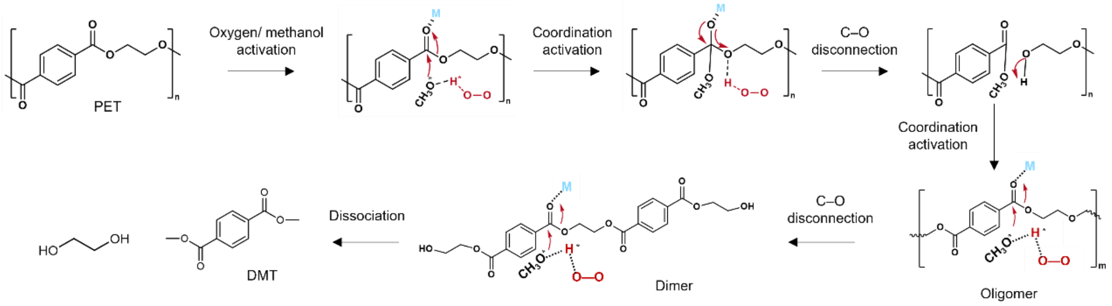

Supplementary Fig. 26. A proposed mechanism of C–O bond cleavage within PET.

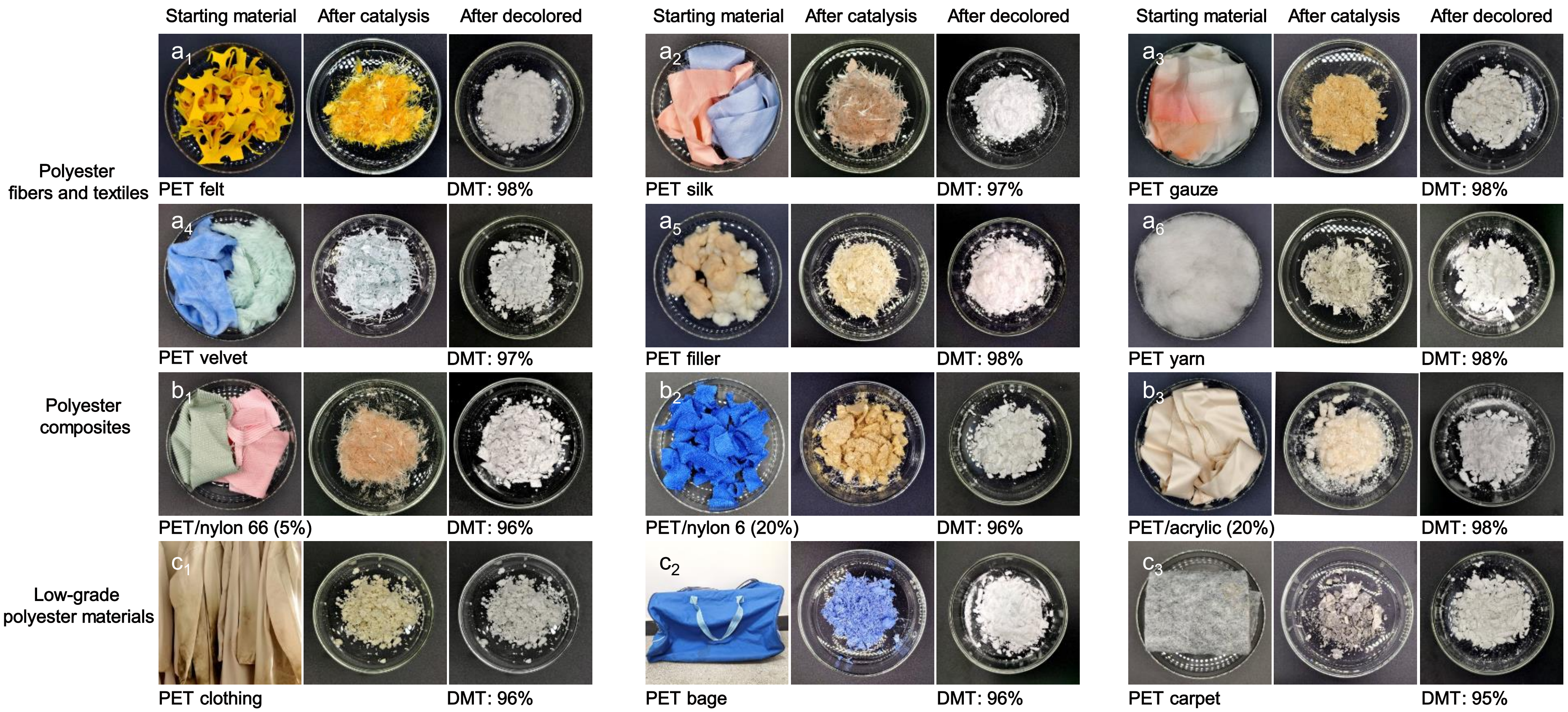

Supplementary Fig. 27. Catalytic depolymerization and recycling of PET from waste consisting of complex components. **a** polyester fibers and textiles; **b** polyester composites; **c** low-grade polyester materials.

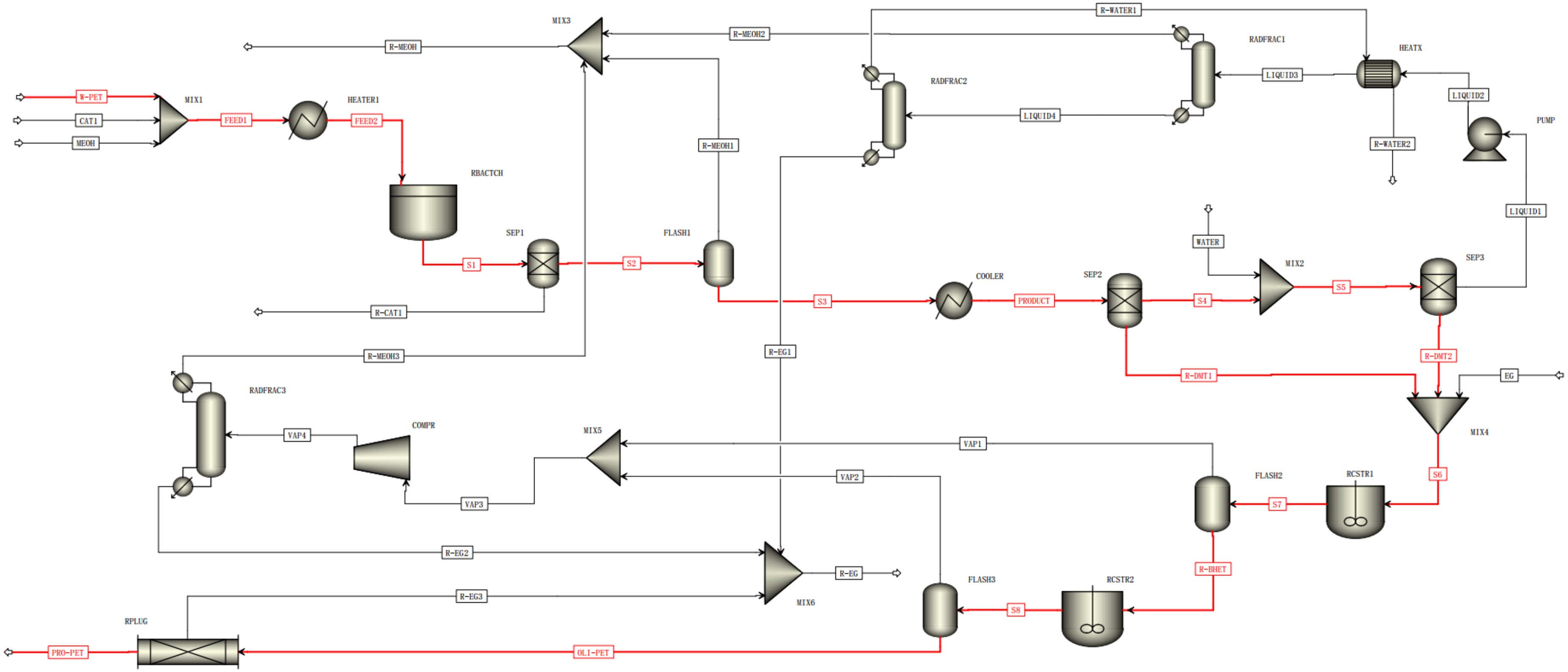

Supplementary Fig. 28. Flowsheet diagram from Aspen model for PET methanolysis and DMT re-polymerization Case.

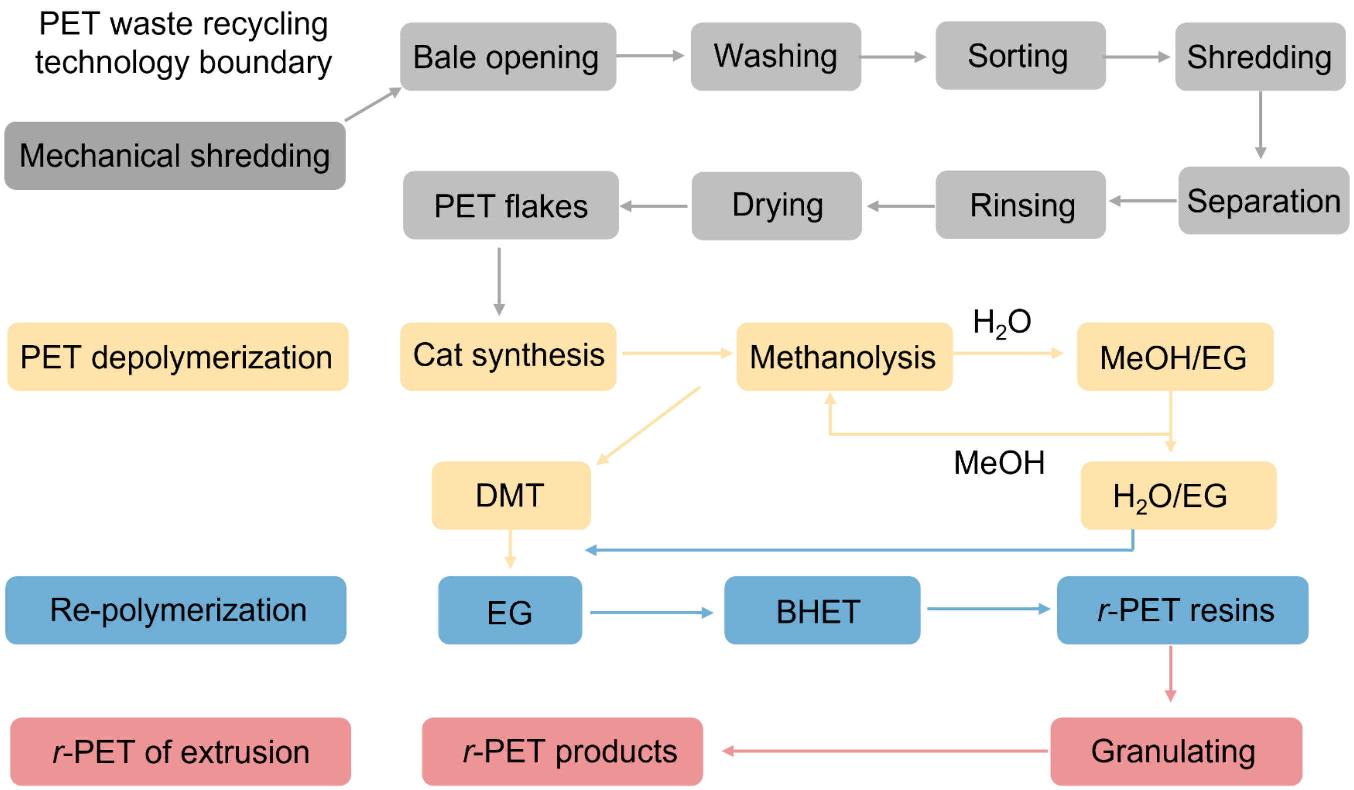

Supplementary Fig. 29. Schematic illustration of closed-loop recycling PET *via* methanolysis route.

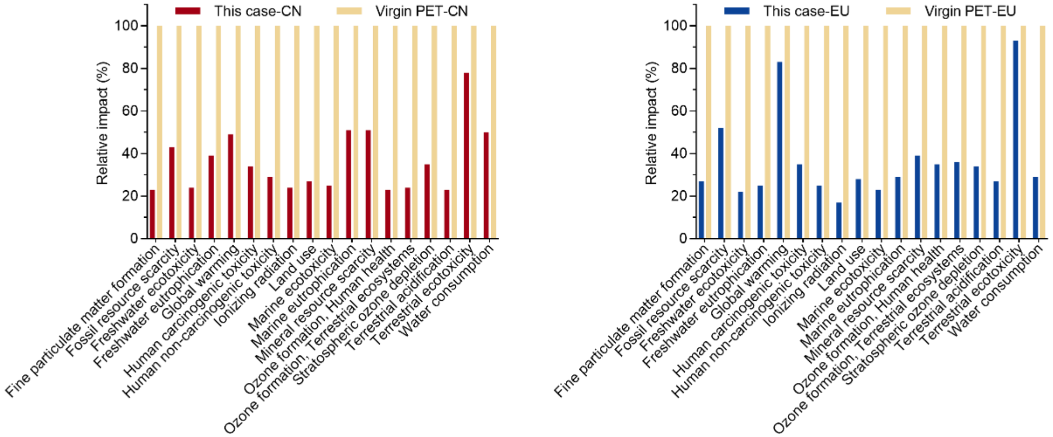

Supplementary Fig. 30. Comparative LCA results of case-CN or case-EU recycling route and virgin PET-CN or virgin PET-EU route.

Supplementary tables

Supplementary Table 1. Comparison of reported catalysts for PET glycolysis.

Entry	Catalyst	Temp. (°C)	$Yield_{BHET}$ (%)[a]	Time (h)	m_{cat}/m_{PET} (mg/g)	STY ($g_{BHET}\cdot g_{cat}^{-1}\cdot h^{-1}$)[b]
1	V_o-rich-ZnO NSs (This work)	180	95.5	40 min	2/1	957.1
2	Solarthermal-Co SSCs[13]	180	82.5	3	0.54/1	675.6
3	CeO_2-2.7 nm[14]	196	90.3	20 min	12.5/1	108.2
4	SO_4^{2-}/CoZnO-300[15]	180	75	3	3/1	109.2
5	SO_4^{2-}-ZnO-TiO_2-200[16]	180	72.8	3	3/1	106.7
6	MgO-Al_2O_3[17]	196	81.3	50 min	10/1	85.2
7	$Zn(OAc)_2$[18]	196	70	1	10/1	84.5
8	MAF-6[19]	180	81.7	4	50/1	26.9
9	Ultrasmall cobalt NPs[20]	180	77	3	15/1	22.6
10	(1,3-DMU)/($Zn(OAc)_2$[21]	190	82	20 min	50/1	18.5
11	Fe_3O_4-MWCNT[22]	190	100	2	50/1	13.2
12	[Ch][OAc] [23]	180	85.2	4	50/1	5.6
13	Solarthermal-CNT-PDA[24]	180	82	2.5	200/1	3.4
14	TBD: MSA[25]	180	92	2	0.25 eq/1 eq	2.9
15	Si-TBD[26]	180	88.5	1.7	15.5 mol%/1 mol	1.2
16	[Bmim][OAc][27]	190	58.2	3	1000/3	0.6
17	Fe_3O_4@SiO_2@(mim)$FeCl_4$[28]	180	100	24	150/1	0.3
18	h-BNNs/Fe_3O_4[29]	200	100	5	2/3	300
19	$ZnMn_2O_4$[30]	260	92.2	1	10/1	92
20	GO-Mn_3O_4[31]	300	96.4	80 min	10/1	64.2
21	γ-Fe_2O_3[32]	300	90	1	50/1	26.4

a Yield of BHET.

b Calculated by: STY $=\frac{m_{BHET}}{m_{cat}\times t}$

Supplementary Table 2. Comparison of reported catalysts for the PET methanolysis.

Entry	Catalyst	Temp. (°C)	$Yield_{DMT}$ (%)[a]	Time/h	m_{cat}/m_{PET} (mg/g)	STY ($g_{DMT}\cdot g_{cat}^{-1}\cdot h^{-1}$)[b]
1	V_o-rich-ZnO NSs (This work)	160	>99	1.0	2/1	505.2
2	V_o-poor-ZnO NSs (This work)	160	2	1.0	20/1	1.0
3	bulk-ZnO NSs (This work)	160	10	1.0	20/1	5.1
4	ZnO nanodispersions[33]	160	80.7	0.5	143/1	11.3
5	bamboo leaf ash[34]	200	78	2.0	100/0.48	1.9
6	Ru–Cu/SiO_2[35]	160	86.5	3.5	20/1	12.5
7	sodium silicate[36]	160	15	0.5	50/1	6.1
8	MgO/NaY[37]	160	10.0	0.5	40/1	5.1
9	Zn(II)-Complexes[38]	150	72.0	1.0	80/1	9.1
10	$[La(acac)_3]$[39]	150	94	4.0	22/1	10.8
11	PIL-Zn^{2+}[40]	160	45	1.0	20/1	22.8
12	[c]$Zn(HMDS)_2$[41]	110	80.0	12.0	50/1	1.3
13	[d]$[NMe_4]^+[OCO_2Me]^-$[42]	100	72	16.0	40/1	1.1
14	[e]K_2CO_3[43]	25	93.1	24.0	200/1	0.2
15	Aluminium triisopropoxide[44]	160	0.8	2.0	500/1	0.0002
16	[f]PET-Zn^{2+} methanol vapor-assisted [45]	160	/	/	0.1/1	662

a Yield of DMT.

b Calculated by: STY $=\frac{m_{DMT}}{m_{cat}\times t}$.

c methanol and toluene as a solvent, M/M=1/1.

d methanol and toluene as a solvent, V/V=1/1.

e methanol and DCM as a solvent, V/V=1/4.

f STY=662 $g_{PET}\cdot g_{cat}^{-1}\cdot h^{-1}$

Supplementary Table 3. Optimization of catalytic methanolysis of PET.

Entry	Temp (°C)	Time (h)	m_{cat}/m_{PET} (mg/g)	PET Conv. (%)	DMT Yield (%)[a]
1	120	1	2/1	0	0
2	130	1	2/1	0	0
3	140	1	2/1	40.5	35
4	150	1	2/1	80.7	75.8
5	160	1	2/1	>99	99
6	170	1	2/1	>99	99
7	180	1	2/1	>99	99
8	200	1	2/1	>99	99
9	160	10 min	2/1	41.5	30.8
10	160	20 min	2/1	62.3	46.3
11	160	30 min	2/1	98	92
12	160	2	2/1	>99	98
13	160	3	2/1	>99	98
14	160	4	2/1	>99	98
15	160	6	2/1	>99	98
16	160	1	0/1	2	0
17	160	1	1/1	98	95
18	160	1	2/1	>99	99
19	160	1	5/1	>99	99
20	160	1	10/1	>99	99
21	120	24	2/1	0	0
22[b]	160	3	2/1	>99	99
23[c]	160	1	2/1	>99	98

a Yield of DMT.

b Experimental conditions for PET methanol amplification depolymerization: PET (40 g), methanol (400 mL), catalyst (80 mg), reaction temperature (160 °C), reaction time (3 h)

c Experimental result of PET methanolysis after 5 cycles over V_o-rich Fe/ZnO NSs.

Supplementary Table 4. The Gibbs free energy (eV) for top and second slabs of ZnO (100) surface.

Reaction	Reaction equation	ΔE_a
Top	ZnO (100) → V_o-ZnO (100) + 1/2 O_2	3.555
Second	ZnO (100) → V_o-ZnO (100) + 1/2 O_2	4.035

Supplementary Table 5. The Gibbs free energy (eV) for different slabs of Fe/ZnO (100) surface.

Reaction	Reaction equation	D1	D2	D3	D4	D5	D6	D7	D8	D9	D10	D11
Thermal desorption	Fe/ZnO (100) → V_o-Fe/ZnO (100) + 1/2 O_2	3.975	3.975	5.135	3.555	3.535	4.025	4.035	4.035	4.015	3.905	3.995

Supplementary Table 6. The Gibbs free energy (eV) for O_2 and methanol of adsorption and activation on bulk ZnO slab.

Reaction	O_2^*	$O_2^*+CH_3OH^*$	TS_1	$V_o^*+OOH^*+CH_3OH^*$
Thermal desorption	-2.27	-3.13	-2.92	-4.78

Supplementary Table 7. The Gibbs free energy (eV) for O_2 and methanol of adsorption and activation on V_o-Fe/ZnO slab.

Reaction	O_2^*	$O_2^*+CH_3OH^*$	TS_1	$V_o^*+OOH^*+CH_3OH^*$
Thermal desorption	-2.32	-3.69	-3.68	-3.64

Supplementary Table 8. The Gibbs free energy (eV) for depolymerization of **Mode 1** into DMT and ethylene glycol on bulk ZnO slabs.

Reaction	R_1	TS_1	M_1	R_2	TS_2	M_2	M_3
Mode 1 depolymerization	0.00	0.08	-1.31	0.00	0.28	0.27	-3.18

Supplementary Table 9. The Gibbs free energy (eV) for depolymerization of **Mode 1** into DMT and ethylene glycol on V_o-Fe/ZnO slabs.

Reaction	R_1	TS_1	M_1	R_2	TS_2	M_2	M_3
Mode 1 depolymerization	0.00	-0.12	-0.42	0.00	-0.40	-0.35	-3.16

Supplementary Note 1. Process descriptions for PET methanolysis and re-polymerization in ASPEN.

The poly-non-random two-liquid (NRTL) physical property method was selected in this simulation to deal with the PET depolymerization and polymerization process (RBACTCH, CSTR1-2, RPLUG) and distillation process (RADFRAC 1-3).

Case (Supplementary Fig. 15): Waste PET was mixed with methanol and subsequently introduced into an intermittent reaction kettle (RBACTCH). The depolymerization reaction was carried out at 160 °C for 1 h. After the reaction, the mixture underwent a filtration process to remove solid catalysts. Since the temperature of the reaction liquid after depolymerization is much greater than the boiling point of methanol, approximately one-third of the methanol was separated through the flash tank (FLASH1). Upon the cooling of the reaction solution, most R-DMT1 crystals precipitate and were subsequently filtered out. Then, water was introduced to the reaction solution to ensure complete crystallization and separation of R-DMT2. The remaining liquid phase was then processed through a series of rectification columns (RADFRAC1-2) for further separation of constituents, including methanol, ethanediol, and water. In these columns (RADFRAC1-2), components were separated based on their boiling points, in the order of ethanediol, water, and methanol. Gaseous methanol was extracted near the top of the first distillation column (RADFRAC1), while gaseous water was separated near the top of the second distillation column (RADFRAC2). High boiling point ethanediol was collected near the bottom of the RADFRAC2 distillation column. After the distillation process, high-purity ethanediol, water, and methanol can be obtained for further reuse. To enhance energy efficiency, the water distillate from RADFRAC2 directly enters the heat exchanger (HEATX) without cooling to fully exchange heat with the low-temperature remaining liquid from the solid-liquid separator (SEP3). This innovative approach significantly bolstered the energy efficiency of the separation process and the chemical raw material utilization efficiency in the entire process, thereby enhancing the overall sustainability of the PET recycling operation.

The re-polymerization stage started with the mixing of ethylene glycol (EG) with recycled dimethyl terephthalate (*r*-DMT) in an all-mixing reactor (RCSTR1). This step involved the transesterification process for creating the PET polyester. The final phase of the re-polymerization involved the production of high molecular weight PET polyester, referred to as PRO-PET. This high-grade polyester was obtained through a high vacuum operation in the post-polymerization reactor (RPLUG), which operated under specific conditions of 270 °C and 1 mm Hg. This recycling of ethylene glycol not only optimizes the use of materials but also enhances the overall sustainability of the process.

Supplementary Note 2. Goal and scope of life cycle assessment (LCA).

LCA in this study, detailed in Supplementary Table 11, evaluates the environmental impact of PET recycling. Besides, the system boundary of LCA was defined as “cradle to gate”, including collection and transportation of waste PET, production of catalysts, and stages of post-depolymerization and re-polymerization. It is assumed that the pretreatment process for PET waste plastic aligns with the established protocols of mechanical recycling. In terms of allocation, our study adopts the “cut-off” rule. This principle delineates the original plastic's lifecycle as distinct and separate from that of the recycled plastic, ensuring an independent evaluation of each lifecycle. The material and energy balance were derived from actual experimental data coupled with Aspen simulation. These form the basis of our “prospective processes”. The functional unit for this LCA is defined as 1 kg of amorphous PET resin (PRO-PET in Aspen Plus).

Impact assessment methodology.

The impact assessment was carried out using the professional software OpenLCA 1.10.3. The assessment methods were IPCC 2021 for GWP and Cumulative Energy Demand for NREU. “Background process” data were sourced from the database Ecoinvent V.3.7.1. Detailed information on the carbon footprint associated with electricity, steam, cooling water, and chemical raw materials, along with their respective reference sources is provided in Supplementary Table 10.

A critical focus of our LCA study is on the greenhouse effect and achieving carbon reduction. Here, we mainly focused on evaluating the NREU (expressed as MJ per kg amorphous PET resin) and GWP (expressed as kg CO_2 equivalent per kg amorphous PET resin, namely kg $CO_{2\text{-eq}}$ /kg amorphous PET resin).

Supplementary Table 10. Goal and scope of this LCA study.

Goal	
Reason and scope	1. Focus on carbon dioxide emissions and consumption of non-renewable energy.<br>2. To assess the GWP and NREU of PET bottles methanolysis, and compare it with published commercial depolymerization protocols in Europe or China (Fig. 6 in main text)
Audience	Industrial stakeholders, the research community, and the public
Application	Provide technical support for polyester plastic carbon emission reduction policies and circular economy
Intention to use results in comparative studies	Yes, the results are to be compared and disclosed to the public through this article's publication
Scope	
Product system	The PET waste depolymerization section with the recycled amorphous PET resin production is based on EU and CN.
Functional unit	1 kg of amorphous PET resin
System boundary	Cradle-to-factory gate, See Fig. 6a in the main text
Allocation	Waste PET cut-off, all environmental effects are allocated to amorphous PET resin chips.
Assumptions	(I) The pre-treatment discharge of waste PET is consistent with the mechanical method<br>(II) This system deals with 25, 000 kg waste PET/per hour over 8,000 hours/per year<br>(III) This system is located in Europe or China<br>(IV) Status-quo technology as of 2020 in the background.
Requirements on data and quality	Foreground material and energy consumption data were obtained from simulation in Aspen Plus and the background processes were chosen based on Ecoinvent V.3.7.1 in Open LCA 1.10.3.
LCIA methodology	IPCC 2021 for GWP; Cumulative Energy Demand for NREU;
Impact categories assessed	1. GWP, 100a, kg CO_2 equivalent;<br>2. NREU, MJ;
Limitations	In addition to the above-mentioned assumptions, the following aspects are not assessed: plant construction and equipment maintenance.
Report requirements	To present the outcome *via* journal publication which is openly accessible to everyone.

Supplementary Table 11. NREU values of each raw material of post-consumer PET bottles methanolysis used in the Open LCA.

Process	Value	Unit	Location
Zinc chloride production	20.42716	MJ per kg	Europe
	35.60782		Rest of the world
Ferric chloride hexahydrate production	17.14334	MJ per kg	Europe
	30.33172		Rest of the world
L-alanine production	15.85558	MJ per kg	Europe without Switzerland
	29.76212		Rest of the world
Market for sodium hydroxide, without water, in 50% solution state	17.64594	MJ per kg	Global of the world
Market for ethanol, without water, in 99.7% solution state, from ethylene	45.49327	MJ per kg	Europe
	52.49459		Rest of the world
Ultrapure water production	0.05960	MJ per kg	Europe
	0.07874		Rest of the world
Market for tap water	0.00617	MJ per kg	Europe without Switzerland
	0.01534		Rest of the world
Market for ethylene glycol	53.00610	MJ per kg	Global of the world
Market group for electricity, low voltage	9.15115	MJ per kWh	Europe without Switzerland
	10.22484		China
Heat production, natural gas, at boiler modulating	1.13333	MJ per MJ	without Switzerland
	1.13628		Rest of the world
Transport, freight, lorry >32 metric ton	1.49960	MJ per t*km	Europe
	1.52019		Rest of the world

Supplementary Table 12. GWP values of each raw material of post-consumer PET bottles methanolysis used in the Open LCA.

Process	Value	Unit	Location
Zinc chloride production	1.31218	kg $CO_{2\text{-eq}}$ per kg	Europe
	2.99507		Rest of the world
Ferric chloride hexahydrate production	1.16427	kg $CO_{2\text{-eq}}$ per kg	Europe
	2.54699		Rest of the world
L-alanine production	1.01292	kg $CO_{2\text{-eq}}$ per kg	Europe without Switzerland
	2.33351		Rest of the world
Market for sodium hydroxide, without water, in 50% solution state	1.28572	kg $CO_{2\text{-eq}}$ per kg	Global of the world
Market for ethanol, without water, in 99.7% solution state, from ethylene	1.18008	kg $CO_{2\text{-eq}}$ per kg	Europe
	1.84898		Rest of the world
Ultrapure water production	0.00300	kg $CO_{2\text{-eq}}$ per kg	Europe
	0.00606		Rest of the world
Market for tap water	0.00034	kg $CO_{2\text{-eq}}$ per kg	Europe without Switzerland
	0.00106		Rest of the world
Market for ethylene glycol	2.01648	kg $CO_{2\text{-eq}}$ per kg	Global of the world
Market group for electricity, low voltage	0.41694	kg $CO_{2\text{-eq}}$ per kWh	Europe without Switzerland
	1.05271		China
Heat production, natural gas, at boiler modulating	0.06964	kg $CO_{2\text{-eq}}$ per MJ	Europe without Switzerland
	0.07065		Rest of the world
Transport, freight, lorry >32 metric ton	0.08599	kg $CO_{2\text{-eq}}$ per t*km	Europe
	0.08889		Rest of the world

Supplementary Table 13. Cradle-to-factory gate LCA results in methanolysis of post-consumer PET bottles, functional unit = 1 kg amorphous PET resin.

Section	China		Europe	
	NREU	GWP	NREU	GWP
Mechanical shredding	5.77469	0.45245	5.51177	0.30259
Catalyst synthesis	7.25578	0.36589	6.32345	0.21044
PET methanolysis	20.82572	1.29502	20.77141	1.27633
Re-polymerization	2.58572	0.16171	2.57666	0.15801
Extrusion	4.90742	0.49151	4.42660	0.20705
Total	41.34934	2.76657	39.60988	2.15442

Units: NREU: MJ/kg amorphous PET resin; GWP: kg $CO_{2\text{-eq}}$/kg amorphous PET resin.

Supplementary Table 14. Material and energy input-output of Mechanical shredding PET transparent bottles, functional unit = 1 kg PET flakes.

Items	Quantity	Unit
Input:		
Waste transparent PET bottles	1088	kg
Transportation distance	400	km
Electricity, low voltage	229.8	kWh
Heat (from natural gas)	2066.8	MJ
Output:		
By-products (e.g., bottle caps, labels)	88	kg
PET flakes	1000	kg

Notes:
CN: NREU 5.77469 MJ / kg PET flakes; GWP 0.45245 kg $CO_{2\text{-eq}}$ / kg PET flakes
EU: NREU 5.51177 MJ / kg PET flakes; GWP 0.30259 kg $CO_{2\text{-eq}}$ / kg PET flakes
All environmental effects are assigned to PET flakes.

Supplementary Table 15. Mass balance of post-consumer PET bottles methanolysis.

Para.	W-PET	CAT1	MEOH	FEED1	FEED2	S1	R-CAT1	S2
Temp./°C	25.00	25.00	69.00	65.07	160.00	160.00	160.00	160.00
Press/kPa	101.33	101.33	126.66	101.33	1714.42	1714.42	1714.42	1714.42
Waste PET (kg/h)	25000.00	0.00	0.00	25000.00	25000.00	0.00	0.00	0.00
Water (kg/h)	0.00	0.00	0.00	0.00	0.00	0.00	0.00	0.00
EG (kg/h)	0.00	0.00	0.00	0.00	0.00	8074.63	0.00	8074.63
DMT (kg/h)	0.00	0.00	0.00	0.00	0.00	25262.26	0.00	25262.26
Methanol (kg/h)	0.00	0.00	197500.00	197500.00	197500.00	189163.12	0.00	189163.12
Cat. 1 (kg/h)	0.00	250.00	0.00	250.00	250.00	250.00	250.00	0.00
BHET (kg/h)	0.00	0.00	0.00	0.00	0.00	0.00	0.00	0.00
Pro-PET (kg/h)	0.00	0.00	0.00	0.00	0.00	0.00	0.00	0.00

Cat. 1: V_o-rich Fe/ZnO.

Supplementary Table 15. Mass balance of post-consumer PET bottles methanolysis. (continued)

Para.	S3	R-MEOH1	PRODUCT	S4	R-DMT1	WATER	S5	R-DMT2
Temp./°C	77.06	77.06	40.00	40.00	40.00	25.00	34.66	34.66
Press/kPa	151.99	151.99	131.72	111.46	111.46	101.33	101.33	101.33
Waste PET (kg/h)	0.00	0.00	0.00	0.00	0.00	0.00	0.00	0.00
Water (kg/h)	0.00	0.00	0.00	0.00	0.00	50700.00	50700.00	50.70
EG (kg/h)	8058.12	16.51	8058.12	7977.54	80.58	0.00	7977.54	7.98
DMT (kg/h)	25258.32	3.94	25258.32	2475.00	22783.32	0.00	2475.00	1809.58
Methanol (kg/h)	119570.29	69592.82	119570.29	118374.59	1195.70	0.00	118374.59	118.37
Cat. 1 (kg/h)	0.00	0.00	0.00	0.00	0.00	0.00	0.00	0.00
BHET (kg/h)	0.00	0.00	0.00	0.00	0.00	0.00	0.00	0.00
Pro-PET (kg/h)	0.00	0.00	0.00	0.00	0.00	0.00	0.00	0.00

Cat. 1: V_o-rich Fe/ZnO.

Supplementary Table 15. Mass balance of post-consumer PET bottles methanolysis. (continued)

Para.	LIQUID1	LIQUID2	LIQUID3	LIQUID4	R-MEOH2	R-MEOH	R-EG1	R-WATER1
Temp./°C	34.66	34.68	90.10	105.20	65.83	69.74	194.16	101.37
Press/kPa	101.33	151.99	151.99	116.52	106.39	101.33	116.52	106.39
Waste PET (kg/h)	0.00	0.00	0.00	0.00	0.00	0.00	0.00	0.00
Water (kg/h)	50649.30	50649.30	50649.30	50604.37	44.93	94.09	49.78	50554.58
EG (kg/h)	7969.56	7969.56	7969.56	7969.56	0.00	16.71	7969.54	0.03
DMT (kg/h)	665.42	665.42	665.42	665.42	0.00	3.94	665.42	0.00
Methanol (kg/h)	118256.22	118256.22	118256.22	100.57	118155.65	197152.08	0.00	100.57
Cat. 1 (kg/h)	0.00	0.00	0.00	0.00	0.00	0.00	0.00	0.00
BHET (kg/h)	0.00	0.00	0.00	0.00	0.00	0.00	0.00	0.00
Pro-PET (kg/h)	0.00	0.00	0.00	0.00	0.00	0.00	0.00	0.00

Cat. 1: V_o-rich Fe/ZnO.

Supplementary Table 15. Mass balance of post-consumer PET bottles methanolysis. (continued)

Para.	R-WATER2	EG	S6	S7	VAP1	R-BHET	S8	VAP2
Temp./°C	95.00	195.00	133.71	195.00	195.00	195.00	230.00	230.00
Press/kPa	106.39	101.33	101.33	111.46	111.46	111.46	101.33	101.33
Waste PET (kg/h)	0.00	0.00	0.00	0.00	0.00	0.00	0.00	0.00
Water (kg/h)	50554.58	0.00	50.70	50.70	48.79	1.91	1.91	1.57
EG (kg/h)	0.03	18865.00	18953.56	3232.38	2095.25	1137.12	6564.51	2014.34
DMT (kg/h)	0.00	0.00	24592.90	0.29	0.05	0.25	0.25	0.01
Methanol (kg/h)	100.57	0.00	1314.08	9429.97	9260.52	169.45	169.45	150.50
Cat. 1 (kg/h)	0.00	0.00	0.00	0.00	0.00	0.00	0.00	0.00
BHET (kg/h)	0.00	0.00	0.00	32197.90	6.48	32191.42	9960.22	1.79
Pro-PET (kg/h)	0.00	0.00	0.00	0.00	0.00	0.00	16803.81	0.00

Cat. 1: V_o-rich Fe/ZnO.

Supplementary Table 15. Mass balance of post-consumer PET bottles methanolysis. (continued)

Para.	VAP3	VAP4	R-MEOH3	R-EG2	OLI-PET	R-EG3	R-EG	PRO-PET
Temp./°C	200.41	208.29	66.60	198.52	230.00	270.00	196.97	270.00
Press/kPa	101.33	111.46	106.39	116.52	101.33	1.37	101.33	1.37
Waste PET (kg/h)	0.00	0.00	0.00	0.00	0.00	0.00	0.00	0.00
Water (kg/h)	50.36	50.36	49.15	1.21	0.34	0.34	51.33	0.00
EG (kg/h)	4109.59	4109.59	0.19	4109.40	4550.17	6961.89	19040.83	18.99
DMT (kg/h)	0.06	0.06	0.00	0.06	0.24	0.23	665.71	0.01
Methanol (kg/h)	9411.01	9411.01	9403.61	7.40	18.95	18.95	26.35	0.01
Cat. 1 (kg/h)	0.00	0.00	0.00	0.00	0.00	0.00	0.00	0.00
BHET (kg/h)	8.27	8.27	0.00	8.27	9958.43	0.65	8.92	1.30
Pro-PET (kg/h)	0.00	0.00	0.00	0.00	16803.81	0.00	0.00	24329.58

Cat. 1: V_o-rich Fe/ZnO.

Supplementary Table 16. Details of the utilities with post-consumer PET bottles methanolysis.

Unit processes used	Type of utility used	Initial and final state of utility	
		Initial	Final
COOLING	Water	20°C; 1.00 atm; liquid phase	25°C; 1.00 atm; liquid phase
L-STEAM	Low-pressure steam	125°C; 2.29 atm; gaseous phase	124°C; 2.22 atm; gaseous phase
M-STEAM	Medium-pressure steam	175°C; 8.86 atm; gaseous phase	174°C; 8.65 atm; gaseous phase
H-STEAM	High-pressure steam	250°C; 39.2 atm; gaseous phase	249°C; 38.6 atm; gaseous phase
TCO	Thermal conductive oil	280°C; 1.00 atm; liquid phase	250°C; 1.00 atm; liquid phase
ELECT	Electricity		

Supplementary Table 17. Utility of post-consumer PET bottles methanolysis.

Unit processes used	Type of utility used	Quantity	Function
HEATER1	Medium-pressure steam	38756.62 kg/h	Heating
COOLER	Water	805591.56 kg/h	Cooling
PUMP	Electricity	4.13 kWh	Increase the pressure
RADFRAC1	Water	6226779.91 kg/h	Cool the top fraction
	Low-pressure steam	79044.67 kg/h	Heat the tower kettle
RADFRAC2	water	547851.83 kg/h	Cool the top fraction
	High-pressure steam	73797.66 kg/h	Heat the tower kettle
RCSTR1	High-pressure steam	6517.74 kg/h	Heating
RCSTR2	High-pressure steam	11667.40 kg/h	Heating
RPLUG	Thermal conductive oil	147300.73 kg/h	Heating
COMPR	Electricity	52.48 kWh	Increase the pressure
PARFRAC3	Water	312410.88 kg/h	Cool the top fraction
	High-pressure steam	418.60 kg/h	Heat the tower kettle

Supplementary Table 18. Energy balance of post-consumer PET bottles methanolysis.

Equipment	HEATER1	RBACTCH	COOLER	PUMP	RADFRAC1		RADFRAC2	
					Condenser	Reboiler	Condenser	Reboiler
Cost (MJ/h)	78861.10	-44218.34	-16817.34	14.868	-129989.00	173256.00	-11436.80	126882.00

Notes:

PET methanolysis (CN): NREU 20.82572 MJ / kg amorphous PET resin; GWP 1.29502 kg $CO_{2\text{-eq}}$ / kg amorphous PET resin

PET methanolysis (EU): NREU 20.77141 MJ / kg amorphous PET resin; GWP 1.27633 kg $CO_{2\text{-eq}}$ / kg amorphous PET resin

Supplementary Table 18. Energy balance of post-consumer PET bottles methanolysis. (continued)

Equipment	RCSTR1	RCSTR2	RPLUG	COMPR	RADFRAC3	
					Condenser	Reboiler
Cost (MJ/h)	11206.10	20060.40	13257.30	188.928	-6521.91	719.92

Notes:

Re-polymerization (CN): NREU 2.58572 MJ / kg amorphous PET resin; GWP 0.16171 kg $CO_{2\text{-eq}}$ / kg amorphous PET resin

Re-polymerization (EU): NREU 2.57666 MJ / kg amorphous PET resin; GWP 0.15801 kg $CO_{2\text{-eq}}$ / kg amorphous PET resin

Supplementary Table 19. Material and energy input-output of Extrusion.

Items	Quantity	Unit
Input:		
PET resin	1031	kg
electricity, low voltage	447	kwh
Heat (from natural gas)	252	MJ
Output:		
amorphous PET resin in forms of chips	1000	kg

Notes:

CN: NREU 4.90742 MJ / kg amorphous PET resin in forms of chips; GWP 0.49151 kg $CO_{2\text{-eq}}$ / kg amorphous PET resin in forms of chips

EU: NREU 4.42660 MJ / kg amorphous PET resin in forms of chips; GWP 0.20705 kg $CO_{2\text{-eq}}$ / kg amorphous PET resin in forms of chips

Supplementary Note 3. Techno-economic analysis (TEA) assumptions

To investigate the economic feasibility of this process for PET cycling, we carried out a simplified techno-economic analysis using a model adapted from that of Aspen simulation and literature reported by Dionisios G. Vlachos's group[46]. The processing capacity of the plant is 200000 tons of waste PET per year. Supplementary Table 19 summarize the model used to calculate the plant-gate levelized cost of processing PET ($/ton PET). The prices of input chemicals and products are listed in the Supplementary. Tables 20-21.

List of assumptions made for the calculations:

1. The capacity factor is expected to be operational on any given day and is assumed to be 0.9, which means the plant will be operational 21.9 hours per day.

2. In the depolymerization stage of waste PET, input chemicals include PET, catalyst, methanol, and water. The output products include *r*-DMT and EG.

3. During the polymerization stage of *r*-DMT, input chemicals include *r*-DMT, catalyst, and EG. The output products include *r*-PET.

4. The capital costs of methanolysis and distillation equipment are dependent on the processing capacity of PET.

5. In this case, the total catalyst cost is just the initial cost of feeding, and the subsequent cycle process is almost negligible due to its low loading, short residence time, and efficient separation of the heterogeneous V_o-rich Fe/ZnO NSs catalyst.

6. Both operation and maintenance costs are based on actual usage and unit price.

7. Utility cost is based on the unit price of public works simulated by aspen software.

8. Transportation cost is based on shipping distance and price.

9. The clean PET flakes could be obtained at $0.66/kg based on the average mixed bale PET cost in recent years. The price of PET waste ($0.39/kg) was assumed to be 40% of virgin PET. (https://jiage.molbase.cn/hangqing/PET) The PET waste textiles could be obtained at $0.13/kg (https://www.chinacace.org/news/view?id=14859).

Supplementary Table 20. Total capital cost, total operating cost, and unit production cost of clear PET, PET wastes, and textile waste cycling.

Total capital cost		Cost (million $)		
Total direct costs	18.12	Equipment Cost	6.45	
		Installed Cost	11.67	
Total indirect costs	4.13	Construction expenses	2.58	
		Legal expenses	0.26	
		Contingency	1.29	
Working capital		3.93		
Total capital cost		26.17		
Total operating cost		Cost (million $ per year)		
/		Clean PET	PET waste	Textile waste
Total catalyst cost		35.12	35.12	35.12
Total transportation cost		5.12	5.12	5.12
Total raw materials		132.00	78.00	20.00
Total utilities cost		19.30	19.30	19.30
Operating labor cost		0.90	0.90	0.90
Maintenance cost		0.32	0.32	0.32
Plant overhead		0.12	0.12	0.12
Total operating cost		192.90	138.90	80.90
Total production cost		Cost (million $ per year)		
/		Clean PET	PET waste	Textile waste
Total Operating Cost		192.90	138.90	80.90
Equipment depreciation expense		1.81	1.81	1.81
Total production cost		194.71	140.71	82.71
Unit production cost [$/t]		1000	723	425

Supplementary Table 21. Total equipment and installed cost of PET cycling.

Items	Equipment Cost [$]	Installed Cost [$]
Grinder	8000	16000
HEATER	128000	290400
RBACTCH	335200	550700
SEP1	99400	286500
FLASH1	61800	220400
COOLER	52900	175800
SEP2	36600	207100
SEP3	40800	213800
PUMP	13700	72700
HEATX	339800	587100
RADFRAC1	1653500	3149400
RADFRAC2	1087300	2359500
RCSTR1	1092210	950830
FLASH2	24300	149700
RCSTR2	1116520	1044180
FLASH3	25900	205900
RPLUG	93800	327800
COMPR	27400	145400
RADFRAC3	200300	700300
Granulator	10000	20000
Total	6447430	11673510

Supplementary Table 22. Utility operation cost of PET cycling.

Items	Utility Cost [$ per year]
water	2794412
Electricity	10526123
Low-pressure steam	2633491
Medium-pressure steam	1387938
High-pressure steam	4477937
Total	19304931

Supplementary References.

1. Kresse, G. & Furthmüller, J. Efficiency of ab-initio total energy calculations for metals and semiconductors using a plane-wave basis set. *Comp. Mater. Sci.* **6**, 15-50 (1996).
2. Surendranath, Y., Kanan, M. W. & Nocera, D. G. Mechanistic Studies of the Oxygen Evolution Reaction by a Cobalt-Phosphate Catalyst at Neutral pH. *J. Am. Chem. Soc.* **132**, 16501-16509 (2010).
3. Perdew, J. P., Burke, K. & Ernzerhof, M. Generalized Gradient Approximation Made Simple. *Phys. Rev. Lett.* **77**, 3865-3868 (1996).
4. Grimme, S., Antony, J., Ehrlich, S. & Krieg, H. A consistent and accurate ab initio parametrization of density functional dispersion correction (DFT-D) for the 94 elements H-Pu. *J. Chem. Phys.* **132**, 154104 (2010).
5. Schleife, A., Rödl, C., Fuchs, F., Furthmüller, J. & Bechstedt, F. Optical and energy-loss spectra of MgO, ZnO, and CdO from ab initio many-body calculations. *Phys. Rev. B* **80**, 035112 (2009).
6. Shoaib Mohammed, Y., Yan, Y., Wang, H., Li, K. & Du, X. Stability of Ferromagnetism in Fe, Co, and Ni Metals under High Pressure with GGA and GGA+U. *J. Magn. Magn. Mater.* **322**, 653-657 (2010).
7. Xu, C. et al. Photothermal Coupling Factor Achieving CO_2 Reduction Based on Palladium-Nanoparticle-Loaded TiO_2. *ACS Catalysis* **8**, 6582-6593 (2018).
8. Henkelman, G., Uberuaga, B. P. & Jónsson, H. A climbing image nudged elastic band method for finding saddle points and minimum energy paths. *J. Chem. Phys.* **113**, 9901-9904 (2000).
9. Henkelman, G. & Jónsson, H. Improved tangent estimate in the nudged elastic band method for finding minimum energy paths and saddle points. *J. Chem. Phys.* 113, 9978-9985 (2000).
10. Wang, J. et al. UiO-66(Zr/Ti) for catalytic PET polycondensation. *Mol. Catal.* **532**, 112741 (2022).
11. Kaiho, S., Hmayed, A. A. R., Delle Chiaie, K. R., Worch, J. C. & Dove, A. P. Designing Thermally Stable Organocatalysts for Poly(ethylene terephthalate) Synthesis: Toward a One-Pot, Closed-Loop Chemical Recycling System for PET. *Macromolecules* **55**, 10628-10639 (2022).
12. Najmi, S., Vance, B. C., Selvam, E., Huang, D. & Vlachos, D. G. Controlling PET oligomers vs monomers via microwave-induced heating and swelling. *Chem. Eng. J.* **471**, 144712 (2023).
13. Liu, Y. et al. Photothermal Catalytic Polyester Upcycling over Cobalt Single-Site Catalyst. *Adv. Funct. Mater.* **33**, 2210283 (2022).
14. Yun, L.-X., Wu, H., Shen, Z.-G., Fu, J.-W. & Wang, J.-X. Ultrasmall CeO_2 Nanoparticles with Rich Oxygen Defects as Novel Catalysts for Efficient Glycolysis of Polyethylene Terephthalate. *ACS Sustainable Chem. Eng.* **10**, 5278-5287 (2022).
15. Zhu, M., Li, Z., Wang, Q., Zhou, X. & Lu, X. Characterization of Solid Acid Catalysts and Their Reactivity in the Glycolysis of Poly(ethylene terephthalate). *Ind. Eng. Chem. Res.* **51**, 11659-11666 (2012).
16. Zhu, M., Li, S., Li, Z., Lu, X. & Zhang, S. Investigation of solid catalysts for glycolysis of polyethylene terephthalate. *Chem. Eng. J.* **185-186**, 168-177 (2012).
17. Chen, F., Wang, G., Li, W. & Yang, F. Glycolysis of Poly(ethylene terephthalate) over Mg–

Al Mixed Oxides Catalysts Derived from Hydrotalcites. *Ind. Eng. Chem. Res.* **52**, 565-571 (2012).

18. López-Fonseca, R., Duque-Ingunza, I., de Rivas, B., Arnaiz, S. & Gutiérrez-Ortiz, J. I. Chemical recycling of post-consumer PET wastes by glycolysis in the presence of metal salts. *Polym. Degrad. Stabil.* **95**, 1022-1028 (2010).
19. Yang, R.-X. et al. Heterogeneous Metal Azolate Framework-6 (MAF-6) Catalysts with High Zinc Density for Enhanced Polyethylene Terephthalate (PET) Conversion. *ACS Sustainable Chem. Eng.* **9**, 6541-6550 (2021).
20. Veregue, F. R. et al. Ultrasmall Cobalt Nanoparticles as a Catalyst for PET Glycolysis: A Green Protocol for Pure Hydroxyethyl Terephthalate Precipitation without Water. *ACS Sustainable Chem. Eng.* **6**, 12017-12024 (2018).
21. Liu, B., Fu, W., Lu, X., Zhou, Q. & Zhang, S. Lewis Acid–Base Synergistic Catalysis for Polyethylene Terephthalate Degradation by 1,3-Dimethylurea/$Zn(OAc)_2$ Deep Eutectic Solvent. *ACS Sustainable Chem. Eng.* **7**, 3292-3300 (2018).
22. Al-Sabagh, A. M., Yehia, F. Z., Harding, D. R. K., Eshaq, G. & ElMetwally, A. E. Fe_3O_4-boosted MWCNT as an efficient sustainable catalyst for PET glycolysis. *Green. Chem.* **18**, 3997-4003 (2016).
23. Liu, Y. et al. Degradation of poly(ethylene terephthalate) catalyzed by metal-free choline-based ionic liquids. *Green. Chem.* **22**, 3122-3131 (2020).
24. Liu, Y. et al. Solar thermal catalysis for sustainable and efficient polyester upcycling. *Matter* **5**, 1305-1317 (2022).
25. Jehanno, C. et al. Organocatalysed depolymerisation of PET in a fully sustainable cycle using thermally stable protic ionic salt. *Green. Chem.* **20**, 1205-1212 (2018).
26. Fehér, Z. et al. Optimisation of PET glycolysis by applying recyclable heterogeneous organocatalysts. *Green. Chem.* **24**, 8447-8459 (2022).
27. Al-Sabagh, A. M. et al. Glycolysis of Poly(ethylene terephthalate) Catalyzed by the Lewis Base Ionic Liquid [Bmim][OAc]. *Ind. Eng. Chem. Res.* **53**, 18443-18451 (2014).
28. Cano, I. et al. Paramagnetic ionic liquid-coated $SiO_2@Fe_3O_4$ nanoparticles—The next generation of magnetically recoverable nanocatalysts applied in the glycolysis of PET. *Appl. Catal. B Environ.* **260**, 118110 (2020).
29. Nabid, M. R., Bide, Y. & Jafari, M. Boron nitride nanosheets decorated with Fe_3O_4 nanoparticles as a magnetic bifunctional catalyst for post-consumer PET wastes recycling. *Polym. Degrad. Stabil.* **169**, 108962 (2019).
30. Imran, M. et al. Manganese-, cobalt-, and zinc-based mixed-oxide spinels as novel catalysts for the chemical recycling of poly(ethylene terephthalate) via glycolysis. *Polym. Degrad. Stabil.* **98**, 904-915 (2013).
31. Park, G. et al. One-step sonochemical synthesis of a graphene oxide–manganese oxide nanocomposite for catalytic glycolysis of poly(ethylene terephthalate). *Nanoscale* **4**, 3879–3885 (2012).
32. Bartolome, L. et al. Superparamagnetic γ-Fe_2O_3 nanoparticles as an easily recoverable catalyst for the chemical recycling of PET. *Green. Chem.* **16**, 279-286 (2014).
33. Du, J.-T. et al. ZnO nanodispersion as pseudohomogeneous catalyst for alcoholysis of polyethylene terephthalate. *Chem. Eng. Sci.* **220**, 115642 (2020).
34. Laldinpuii, Z. T. et al. Methanolysis of PET Waste Using Heterogeneous Catalyst of Bio-

waste Origin. *J. Polym. Environ.* **30**, 1600-1614 (2021).

35. Tang, H. et al. Synthesis of gasoline and jet fuel range cycloalkanes and aromatics from poly(ethylene terephthalate) waste. *Green. Chem.* **21**, 2709-2719 (2019).

36. Tang, S. et al. Calcined sodium silicate as solid base catalyst for alcoholysis of poly(ethylene terephthalate). *J. Chem. Technol. Biot.* **97**, 1305-1314 (2022).

37. Tang, S. et al. MgO/NaY as modified mesoporous catalyst for methanolysis of polyethylene terephthalate wastes. *J. Environ. Chem. Eng.* **10**, 107927 (2022).

38. Payne, J. M., Kociok-Köhn, G., Emanuelsson, E. A. C. & Jones, M. D. Zn(II)- and Mg(II)-Complexes of a Tridentate Ligand: Application to Poly(lactic acid) Production and Chemical Upcycling of Polyesters. *Macromolecules* **54**, 8453-8469 (2021).

39. Abe, R., Komine, N., Nomura, K. & Hirano, M. La(iii)-Catalysed degradation of polyesters to monomers via transesterifications. *Chem. Commun.* **58**, 8141-8144 (2022).

40. Jiang, Z. et al. Poly(ionic liquid)s as efficient and recyclable catalysts for methanolysis of PET. *Polym. Degrad. Stabil.* **199**, 109905 (2022).

41. Yang, R., Xu, G., Dong, B., Guo, X. & Wang, Q. Selective, Sequential, and “One-Pot” Depolymerization Strategies for Chemical Recycling of Commercial Plastics and Mixed Plastics. *ACS Sustainable Chem. Eng.* **10**, 9860-9871 (2022).

42. McKeown, P. et al. Organocatalysis for versatile polymer degradation. *Green. Chem.* 22, 3721-3726 (2020).

43. Mjalli, F. S., Naser, J., Jibril, B., Al-Hatmi, S. S. & Gano, Z. S. Ionic liquids analogues based on potassium carbonate. *Thermochim. Acta* **575**, 135-143 (2014).

44. Kurokawa, H., Ohshima, M.-a., Sugiyama, K. & Miura, H. Methanolysis of polyethylene terephthalate (PET) in the presence of aluminium tiisopropoxide catalyst to form dimethyl terephthalate and ethylene glycol. *Polym. Degrad. Stabil.* **79**, 529-533 (2003).

45. Bai, X., Aireddy, D. R., Roy, A. & Ding, K. Solvent-Free Depolymerization of Plastic Waste Enabled by Plastic-Catalyst Interfacial Engineering. *Angew. Chem. Int. Ed.* **62**, e202309949 (2023).

46. Luo, Y., Selvam, E., Vlachos, D. G. & Ierapetritou, M. Economic and Environmental Benefits of Modular Microwave-Assisted Polyethylene Terephthalate Depolymerization. *ACS Sustainable Chem. Eng.* **11**, 4209-4218 (2023).
